# Levels of impulsivity in individuals with cannabis use disorder

**DOI:** 10.47626/2237-6089-2021-0449

**Published:** 2022-06-27

**Authors:** Marcia Fortes Wagner, Camila Rosa de Oliveira, Luís Henrique Paloski

**Affiliations:** 1 Programa de Pós-Graduação em Psicologia Faculdade Meridional IMED Passo Fundo RS Brazil Grupo de Estudos e Pesquisas Relações Interpessoais, Emoção, Comportamento e Cognição, Programa de Pós-Graduação em Psicologia, Faculdade Meridional (IMED), Passo Fundo, RS, Brazil.; 2 Núcleo de Investigação em Cognição, Emoção e Tecnologias em Neuropsicologia e Saúde Programa de Pós-Graduação em Psicologia IMED Passo Fundo RS Brazil Núcleo de Investigação em Cognição, Emoção e Tecnologias em Neuropsicologia e Saúde, Programa de Pós-Graduação em Psicologia, IMED, Passo Fundo, RS, Brazil.; 3 Núcleo de Intervenções e Pesquisas em Avaliação, Saúde e Carreira Programa de Pós-Graduação em Psicologia IMED Passo Fundo RS Brazil Núcleo de Intervenções e Pesquisas em Avaliação, Saúde e Carreira, Programa de Pós-Graduação em Psicologia, IMED, Passo Fundo, RS, Brazil.

**Keywords:** Cannabis use disorder, impulsive behavior, adults

## Abstract

**Introduction:**

Substance use disorder consists of the presence of cognitive, behavioral, and physiological symptoms, indicating continuous use of one or more substances by the individual. The literature points to the existence of a relationship between impulsive behavior, in which individuals tend to act thoughtlessly and with a lower level of planning, and consumption of substances including cannabis.

**Objectives:**

To examine the presence and severity of impulsivity in individuals with cannabis use disorder and investigate associations between sociodemographic and clinical characteristics and impulsivity.

**Method:**

Participants completed a sociodemographic data sheet and the Barratt Impulsiveness Scale (BIS-11). A total of 122 subjects with a diagnosis of cannabis use disorder participated, with a mean age of 34.46 years (standard deviation = 9.62).

**Results:**

The prevalence of high levels of impulsivity in the sample ranged from 30 to 33%; the BIS-11 total score was significantly associated with cohabitation and alcohol use. The BIS-11 scores for motor impulsivity and attentional impulsivity were also associated with consumption of alcohol. No associations were found between impulsivity and the variables age, education, use of tobacco, or use of cocaine/crack.

**Conclusion:**

This study contributes to understanding of substance dependence, especially cannabis. It found presence of impulsive behavior among individuals with cannabis use disorder, which is corroborated by reports in the literature.

## Introduction

Substance use disorder is related to presence of a set of cognitive, behavioral, and physiological symptoms that indicate continuous use by the individual, despite the substance-related problems.^1^ Dependence is increased by the individual’s continuous and long-lasting exposure to the substance, accumulating psychological, neurobiological, and social factors.^1^

According to the United Nations Office for Drugs and Crime (UNODC), the use of *Cannabis sativa*, also known as marijuana or cannabis, has increased in several parts of the world,^2^ ranking it as the third most consumed substance in the world, after alcohol and cigarettes.^2-4^ Cannabis is also the illicit substance most consumed in Brazil and 3.2% of the Brazilian population have used psychoactive substances, which equates to 4.9 million people.^4^Reported rates of use are higher in men than in women and use is more common between 18 and 29 years of age and less common from 65 years onwards.^5^

The literature reports that it is common for individuals who use substances to have comorbidities such as depressive and anxiety disorders, among others, with emphasis on impulsive behaviors.^4-6^ Impulsive behaviors can be considered a broad phenotype with varied and peculiar characteristics, presenting distinct types of behaviors and cognitive expressions.^6^ Despite their multifaceted character, they are identified as rapid behaviors, performed without assessment of the context and linked with difficulty in controlling the response. In turn, impulsiveness is related to such behaviors and impulsive individuals tend to act thoughtlessly and with a lower level of planning.^7^ Impulsiveness negatively affects the individual and people around them^8^ and is associated with use of substances.^9^ An investigation into the impulsive behavior of psychoactive substance-dependent individuals, using the Barratt Impulsiveness Scale (BIS-11),^8^ yielded important results that confirmed a direct relationship between impulsive behavior and substance dependence.^1^

A study with cannabis users showed impairment of self-control of aggression in adverse situations.^10^ Results were supported by another investigation with cannabis users that also revealed impairment of self-control of aggression.^11^ These findings suggest that individuals who consume cannabis face greater difficulty in coping with what they feel, based on the social situations they experience, engaging in substance-seeking behaviors to deal with their impulsivity.

This study aimed to investigate the presence and severity of impulsivity in individuals with cannabis use disorder, to verify to what extent impulsive behaviors may be linked to substance use. Moreover, it aimed to test for associations between sociodemographic and clinical characteristics (age, education, marital status, cohabitation, and use of other psychoactive substances) and impulsivity. This investigation should contribute to elucidating factors associated with impulsivity among individuals with cannabis use disorder, providing data that could assist in planning of more effective intervention strategies for treatment of this clinical condition.

## Method

### Participants and procedure

This is a cross-sectional quantitative study. It recruited 122 subjects with a diagnosis of cannabis use disorder. The sample had a mean age of 34.46 years (standard deviation [SD] = 9.62, minimum = 19 years, maximum = 62 years), 86.1% (n = 105) were male, and 13.9% (n = 17) were female.

Inclusion criteria were age ≥ 18 years and a minimum education level corresponding to the 5th grade of Brazilian elementary school. All participants should meet diagnostic criteria for cannabis use disorder based on the criteria for substance use disorders from the Diagnostic and Statistical Manual of Mental Disorders, 5th version (DSM-5)^5^ and have been admitted to a rehabilitation institution for at least 15 days, after detoxification, the initial stage of hospitalization, in which the individual is encouraged to stop using substances, with the objective of ridding the body of the toxic effects of their ingestion. These changes may be behavioral or psychological, such as impaired judgment, aggression, sudden changes in mood, and disturbances affecting wakefulness, attention, thinking, and psychomotor and interpersonal behavior, among others.^5^Subjects hospitalized for less than 15 days were excluded, as were those with any psychological impairment that would prevent them from understanding the instruments.

Data were collected at institutions specialized in the care of adults with diagnosis of substance use disorder in cities in Rio Grande do Sul state, Brazil. Institutions were previously contacted to authorize the study and data collection was started after authorization was granted. Participants were informed about the nature and purposes of the research, and of the researchers’ commitment to maintaining confidentiality of participants’ identity as well as of the need to sign the informed consent form, pursuant to Resolution 466/12 of the Brazilian National Health Council that regulates research in human beings in Brazil.

### Instruments

The instruments used were as follows: 1) sociodemographic data sheet, to collect the main data on participants; 2) structured clinical interview that covered history of substance use and morbidities. The structured clinical interview was conducted according to the DSM-5^5^ criteria to confirm the diagnosis of dependence; and 3) the BIS-11,^8^ a 30-item instrument on the manifestations of impulsivity, based on Ernst Barratt’s theoretical model, with responses on a four-point Likert scale where 1 = rarely or never; 2 = from time to time; 3 = frequently; 4 = almost always/always. Scoring ranges from 30 to 120 points, and high values indicate the presence of impulsivity. The original BIS-11 was a three-factor scale measuring motor impulsivity, attentional impulsivity, and impulsivity due to lack of planning.^12^ In a Brazilian population, Malloy-Diniz et al.^8^ present normative data for adults aged 18 to 84 years old, allowing determination of occurrence of impulsivity impairments both for the general score and for the three factors of the scale. In this study, the alpha value for the BIS-11 general score was 0.823, while alpha for the motor impulsivity factor was 0.650, alpha for attentional impulsivity was 0.703, and alpha for impulsiveness due to lack of planning was 0.623.

### Ethical approval

The study was approved by the research ethics committee at Faculdade Meridional (IMED; CAAE 43367620.8.0000.5319) and was conducted in accordance with the Declaration of Helsinki. The researchers guarantee that individuals provided written consent and that all documentation will be kept confidential.

### Data analysis

Statistical analysis was conducted using the Statistical Package for the Social Sciences (SPSS v. 25), and Jeffrey’s Amazing Statistics Program (JASP 0.14.1.). Quantitative variables were reported as mean and SD. Occurrence of deficits in BIS-11 (general score and factors) was identified by calculating z scores ([normative group – points obtained]/SD normative group), defining presence of deficit as a z-score ≤ -1.501^13^ and as scores equivalent to ≥ 90 percentiles for reference values from normative groups extracted from the study by Malloy-Diniz et al.^8^ These two forms of classification were selected because they are recognized measures for expressing the relative positions of scores in a reference distribution.^14^ Associations between occurrence of deficits and other sociodemographic and clinical variables were confirmed using the chi-square test.

## Results

Table 1 shows the participants’ sociodemographic data and use of other substances. Prevalence was higher among participants who had only attended elementary school, single individuals, and those living with other individuals (for example: uncles, brothers, and friends). As for the data on substance use, most of the participants also used other substances, such as alcohol, tobacco, and cocaine/crack, in addition to cannabis.

**Table 1:** Sociodemographic data and use of psychoactive substances

	n (%)
Age (years)	
19-29	39 (32)
30-62	83 (68)
Education	
Elementary school	50 (41)
High school	35 (29)
Higher education	37 (30)
Marital status	
Married or in partnership	23 (19)
Separated	23 (19)
Widowed	1 (1)
Single	75 (61)
Cohabitation	
Alone	20 (16)
With husband/wife or partner	7 (6)
With husband/wife or partner and children or grandchildren	5 (4)
Parents	17 (14)
Others*	73 (60)
Use of other substances in addition to cannabis	
Alcohol	92 (75)
Tobacco	79 (65)
Cocaine/crack	108 (89)

* Relatives and friends.

Table 2 presents descriptive statistics for the BIS-11 data in addition to total scores classified by percentiles. For the total BIS-11 score, from 30 to 33% of the participants reported impulsivity levels classified as deficits, based on the z score cutoff of ≤ -1.50, and percentiles greater than or equal to 90, respectively. Regarding factors, and based on the same benchmarks, motor impulsivity was the factor with highest percentage of deficit(37% by z score; 43% by percentile), followed by attentional impulsivity (24% by z score; 26% by percentile), and impulsivity due to lack of planning (21% by z score; 26% by percentile). In relation to factor scores, impulsivity due to lack of planning had the highest score, with a mean of 27.93 (SD = 5.9).

**Table 2:** Means, standard deviations and Barratt Impulsiveness Scale (BIS-11) score classifications

	BIS-11			
	Total	Motor impulsivity	Attentional impulsivity	Impulsivity due to lack of planning
Mean	70.12	23.53	18.66	27.93
Standard deviation	12.90	5.47	4.74	5.49
Minimum	32	13	8	11
Maximum	101	39	30	41
				
Percentile (%)				
< 25	12 (10)	12 (10)	19 (16)	11 (10)
25	19 (16)	13 (11)	13 (11)	24 (19)
50	25 (20)	23 (19)	29 (24)	36 (30)
75	26 (21)	21 (17)	28 (23)	18 (15)
90	30 (25)	43 (35)	31 (25)	24 (19)
99	10 (8)	10 (8)	2 (1)	9 (7)

Table 3 shows the chi-square analysis of associations between sociodemographic variables, substance use (alcohol, tobacco, and cocaine/crack), and presence of impulsivity according to the BIS-11 score and classification based on z scores. The BIS-11 total score was significantly associated with the variable cohabitation and alcohol use. This result suggests that adults who live alone and consume alcohol together with cannabis tend to report higher rates of impulsivity. The scores for the BIS-11 motor impulsivity and attentional impulsivity factors were also significantly associated with alcohol consumption. This suggests that individuals with cannabis use disorder and those who also use alcohol report greater impairment in their ability to inhibit responses and lack of focus when performing tasks. No associations were found with age, education, tobacco use, or cocaine/crack use.

**Table 3:** Chi-square (X2) tests for associations between sociodemographic variables and presence of impulsivity deficits according to the Barratt Impulsiveness Scale (BIS-11)

	Total		Motor		Attentional		Lack of planning	
	X^**2**^	p	X^**2**^	p	X^**2**^	p	X^**2**^	p
Age	0.403	0.525	0.024	0.877	1.072	0.300	0.933	0.334
Education	1.513	0.469	1.377	0.502	3.418	0.181	2.008	0.366
Marital status	4.146	0.246	2.619	0.454	0.727	0.867	2.604	0.457
Cohabitation	12.068	0.017*	4.441	0.350	6.924	0.140	4.384	0.357
Alcohol use	5.004	0.025*	4.872	0.027*	4.163	0.041*	2.688	0.101
Tobacco use	0.297	0.586	1. 262	0.261	0.296	0.587	0.311	0.577
Cocaine/crack use	0.007	0.935	1.168	0.280	1.149	0.284	0.008	0.927

* p < 0.05.

## Discussion

The main objective of this study was to examine presence and severity of impulsivity in individuals diagnosed with substance use disorder, specifically users of cannabis. Therefore, the performance of participants on the BIS-11 scale was validated and normative data for the Brazilian context were classified based on z scores and percentile rankings. The prevalence of high impulsivity scores found in the sample of this study ranged between 30 and 33%, depending on the classification adopted (z score or percentile). When it comes to the BIS-11 factors, the motor impulsivity factor reported the highest percentage of impairment among participants (37% by z score; 43% by percentile). Another objective of this study was to test for associations between sociodemographic and clinical characteristics (age, education, marital status, cohabitation, and use of other psychoactive substances) and impulsivity. Participants who live alone and consume alcohol together with cannabis tend to exhibit more impulsive behaviors. In addition, the occurrence of high levels of motor and attentional impulsivity was also associated with alcohol consumption.

The prevalence of problems of impulsivity in the study sample exceeded the levels expected for the population at large (approximately 17%),^15,16^ although no specific values were found for the clinical population with substance use disorder, mainly related to the use of cannabis. However, high levels of impulsivity were found in college students who use cannabis.^17-19^ This finding supports the results found in literature, according to which presence of impulsive behaviors is very common in several disorders,^5^ such as in substance use disorder.^5,9,16^ It highlights the existence of a direct linkage between impulsivity and dependence on psychoactive substances.^1^

Findings are also in line with other studies that also found impaired self-control of aggression among cannabis users.^10,11^ This result reinforces a trait of impulsivity in individuals with cannabis use disorder and difficulty in dealing with criticism from others. In adverse situations, they may react with poor control of anger and aggression. These behaviors may trigger more impairments in their interpersonal relationships.

In line with this study’s findings, the literature confirms that prevalence of substance use disorder is higher among men.^5^ Despite the higher prevalence of participants in the age group of 30 to 62 years, the mean age found in the sample bears out DSM-5^5^ studies affirming that use among middle-aged adults and the elderly is increasing. Rather et al.^17^ and Goodman et al.^18^ also reported similar ages for the profile of adults using cannabis. In this study, however, there was no association between higher levels of impulsivity and age, although it has been observed that impairments in executive components, including impulsivity, arising from the use of cannabis were more frequent among adolescents in comparison to adults.^19^

Still regarding sociodemographic characteristics, despite evidence that lower education levels are associated with use of substances,^20^ there is no consensus in the literature on this relationship, since other variables may influence these results. For example, Beaton et al.^21^ observed an important effect of age and income when reviewing the association between education and impulsivity among adults with substance use disorders. Therefore, these characteristics appear to interact in a way that could explain the highest occurrence of high levels of impulsivity in that population.

The sample studied had a high rate of users who also use other substances in addition to cannabis, such as alcohol, tobacco, and cocaine/crack. Data from the Brazilian literature suggest there is concomitant use of several substances in several different age groups.^22,23^ Another study with patients hospitalized due to use of substances found that most participants reported using multiple substances such as cannabis, alcohol, and crack/cocaine.^24^

The results of this study suggest that adults living alone and consuming alcohol jointly with cannabis tend to report higher rates of impulsivity. Data in the literature suggest that use of several psychoactive substances is associated with changes in levels of impulsivity^1,25^and can be related to family conflicts.^26^ Therefore, social isolation and feelings of loneliness may be risk factors for development of substance use disorders.^27,28^

## Conclusion

This study contributes to understanding of substance dependence, especially cannabis. High levels of impulsivity were found in most of the sample of individuals with cannabis use disorder, which is corroborated by the literature.

This highlights the importance of conducting studies to carry out comparative analyses of impulsivity in individuals with cannabis use disorder and in the general population, to contribute to existing interventions and to design of appropriate and effective interventions. Professionals who work in treatment of substance users would thereby be better equipped to assist in these individuals’ process of behavioral change, targeting reduction and consequent cessation of substance use, expansion of competence in interpersonal relationships, and better quality of life.

The regional nature of the sample is one perceived limitation and the predominance of men in the sample is another. Furthermore, the study design does not allow cause-and-effect between variables to be assigned and it was not possible to control for covariates in the association analyses because of the heterogeneity of the clinical sample. It is suggested that further investigations should be conducted with larger samples and in different regions of Brazil, as well as new studies investigating the subject.
